# Transcriptome characterization and gene expression of *Epinephelus* spp in endoplasmic reticulum stress-related pathway during betanodavirus infection *in vitro*

**DOI:** 10.1186/1471-2164-13-651

**Published:** 2012-11-21

**Authors:** Ming-Wei Lu, Fang-Huar Ngou, Yung-Mei Chao, Yu-Shen Lai, Nai-Yu Chen, Fan-Yao Lee, Pinwen P Chiou

**Affiliations:** 1 Department of Aquaculture, National Taiwan Ocean University, Keelung, Taiwan; 2 Center of Excellence for Marine Bioenvironment and Biotechnology, National Taiwan Ocean University, Keelung, Taiwan; 3 Institute of Basic Medical Science, National Cheng Kung University, Tainan, Taiwan; 4 Institute of Biotechnology, National Ilan University, Yilan, Taiwan; 5 Marine Research Station, Institute of Cellular and Organismic Biology, Academia Sinica, Yilan, Taiwan

## Abstract

**Background:**

Grouper (*Epinephelus* spp) is an economically important fish species worldwide. However, viral pathogens such as nervous necrosis virus (NNV) have been causing severe infections in the fish, resulting in great loss in the grouper aquaculture industry. Yet, the understanding of the molecular mechanisms underlying the pathogenicity of NNV is still inadequate, mainly due to insufficient genomic information of the host.

**Results:**

*De novo* assembly of grouper transcriptome in the grouper kidney (GK) cells was conducted by using short read sequencing technology of Solexa/Illumina. A sum of 66,582 unigenes with mean length of 603 bp were obtained, and were annotated according to Gene Ontology (GO) and Clusters of Orthologous Groups (COG). In addition, the tag-based digital gene expression (DGE) system was used to investigate the gene expression and pathways associated with NNV infection in GK cells. The analysis revealed endoplasmic reticulum (ER) stress response was prominently affected in NNV-infected GK cells. A further analysis revealed an interaction between the NNV capsid protein and the ER chaperone immunoglobulin heavy-chain binding protein (BiP). Furthermore, exogenous expression of NNV capsid protein was able to induce XBP-1 mRNA splicing *in vivo*, suggesting a role of the capsid protein in the NNV-induced ER stress.

**Conclusions:**

Our data presents valuable genetic information for *Epinephelus* spp*.*, which will benefit future study in this non-model but economically important species. The DGE profile of ER stress response in NNV-infected cells provides information of many important components associated with the protein processing in ER. Specifically, we showed that the viral capsid protein might play an important role in the ER stress response.

## Background

Because of the exquisite quality of flesh, grouper is one of the aquaculture species with high economic value. In past decades, the grouper industry has developed rapidly in many Asian countries. In Taiwan alone, the production volume of grouper was about 9,300 mt in 2006, reaching a total value of NT$ 1.73 billion, approximate US$60 million [1]. However, the sustainable development of grouper industry has been threatened by infection caused by nervous necrosis virus (NNV), which could cause mass mortality of up to 100% in the fish at larval and juvenile stages [2-5].

NNV, also known as viral encephalopathy and retinopathy (VER), belongs to the genus *Betanodavirus* of the family *Nodaviridae*[5]. NNV virion is non-enveloped, spherical-shaped particle with a diameter of 25–34 nm [2,6,7]. It consists of two single-stranded, positive-sense RNA genomes: RNA1 (3.1kb) encodes the viral RNA-dependent RNA polymerase (RdRp) and RNA2 encodes the viral capsid protein [8]. In addition, a subgenomic RNA3 from RNA1 contains two open reading frames that encode protein B1 (111 amino acids) and B2 (75 amino acids), which have been shown to be an anti-necrosis and a suppressor of host siRNA silencing factor, respectively [9,10]. Protein B2 might also function as necrotic death factor [11]. The first NNV outbreak was reported in Japanese parrotfish, *Oplegnathus fasciatus* (Temminck & Schlegel) in 1990 [2], and has been since widely reported in various teleost species, including grouper. In grouper, the infected fish exhibit signs of uncoordinated darting, spiral swimming, abnormalities of swimming bladder control, and vacuolation in brain and retina [2,4,12-16].

NNV infection remains as now a major threat to the grouper industry, despite extensive efforts have been invested to develop therapeutic and prophylactic regiments against the viral infection. Extended understanding of the molecular mechanism of the viral pathogenicity can facilitate the development of antiviral strategy, yet such knowledge is currently inadequate. For examples, the infection route of NNV in the host is not fully illustrated, and the cellular response of the host toward NNV remains largely in puzzle. NNV is known to cause apoptotic death of infected cells. Recent studies have further indicated a connection between the viral-induced endoplasmic reticulum (ER) stress and the apoptotic death [17-19]. Many studies have demonstrated that virus-induced ER stress could determine the fate of infected cell, survival or death [20,21]. Cell which does not overcome the ER stress will succumb to apoptosis ultimately. In the stressed cell, the ER chaperone immunoglobulin heavy-chain binding protein (BiP), also known as glucose-regulated protein 78 (GRP78), functions as a master control to relieve the stress by initiating the unfolding protein response (UPR) pathway. BiP activates the three mediators of UPR: PKR-like ER kinase (PERK), activating transcription factor 6 (ATF6), and inositol-requiring 1 (IRE1). Activation of PERK could phosphrylate eIF-2α, resulting in attenuation of protein synthesis. On the other hand, ATF6 and IRE1 function at the later stage of UPR as compared to PERK. After being translocated into nucleus, the activated ATF6 stimulates the expression of several chaperones that are capable of refolding misfolded proteins. ATF6 also stimulates the expression of the X-box-binding protein (XBP1) mRNA, which would produce a transcription factor once one of its intron is removed by IRE1. The product of the spliced XBP1 mRNA stimulates the expression of proteins that could facilitate the degradation of misfolded proteins [17]. All together, these mediators alleviate ER stress by assisting protein folding, reducing translocation of newly synthesized proteins into ER, and facilitating protein degradation in ER lumen. Currently, the understanding of NNV-induced ER stress response is insufficient and requires further investigation.

One hurdle to the study of the molecular mechanism of NNV pathogenicity in grouper cells is the lack of sufficient genomic information of the host. The recent advance in the next generation sequencing (NGS) technology has exerted profound impact on the biological science research. The technique has been applied in decoding the genomes of several non-model organisms by transcriptome sequencing, providing valuable information in the understanding of gene function, cell responses and evolution. Transcriptome sequencing is cost effective compared to traditional Sanger sequencing method, and is a suitable alternative to whole genome sequencing as it can provide information of transcribed portion of genes at a lower cost [22].

In this study, grouper kidney cell (GK cell) was used to establish a transcriptome library by using the NGS technology. A total of 66,584 unigenes were obtained by SOAP *de novo*[23] transcriptome assembly software from 51 million reads of raw mRNA sequencing data. By matching the assembled unigenes with various databases, we have characterized these unigenes into different functional categories and pathways. The analysis resulted in the identification of 25 unigenes that are relevant to ER stress-associated genes, including BiP, PERK, ATF6, IRE1, and XBP-1. We further found that BiP could interact with the NNV capsid protein in the infected cells, suggesting a role of these two proteins in the ER stress response after viral infection. These data will benefit future study of NNV pathogenicity and development of therapeutic or prophylactic treatment against NNV.

## Results

### *De novo* sequencing and reads assembly

The grouper kidney GK cell line was used in this study to generate a transcriptome database. After eliminating read with adaptors, read with unknown nucleotides larger than 5% and low quality read, 51,198,090 paired-end reads from total nucleotide of 4,607,828,100 were generated by using the SOAP *de novo* transcriptome assembly program. By overlapping reads into longer fragments, 204,517 contigs with mean length of 248bp were combined. The contigs were assembled into 79,678 scaffolds, which were further assembled into a total number of 66,582 unigenes with mean length of 603 bp (Table 1). The sequence length with highest percentage was in the range of 100–1000 bp (84.5%), followed in succession by 1001–2000 bp (10.7%), 2001–3000 bp (3.2%), and longer than 3000 bp (1.6%).

**Table 1:** Summary of the grouper GK cell transcriptome

Total Reads	51,198,090
Total Nucleotides (nt)	4,607,828,100
Total Contig Number	204,517
Mean Length of Contig (bp)	248
Total Contig Length (nt)	50,696,343
Total Scaffold Number	79,687
Mean Length of Scaffold (bp)	527
Total Scaffold Length (nt)	41,984,530
Total Unigene Number	66,582
Mean Length of Unigene (bp)	603
Total Length of all Unigene (nt)	40,146,797

### Gene ontology (GO) and clusters of orthologous groups (COG) annotation of unigenes

The GK transcriptome unigenes were annotated according to two functional annotation conventions, Gene Ontology (GO) and Clusters of Orthologous (COG). The unigenes were classified into the three categories of GO: molecular function, cellular component and biological process, and 51 sub-categories. Because multiple GO functions were assigned to a single unigene in some cases, 76,975 assignments were generated from the 66,582 unigenes. The biological process was the dominant category, which made up of 49.57%, followed by cellular component (35.45%), and molecular function (14.99%) (Additional file 1: Figure S1). A total of 19,261 unigenes were grouped into the 25 COG categories. The largest group in COG is ‘general function prediction only’ (3673, 19.07%), followed by group ‘transcription’ (1708, 8.87%) and group ‘replication, recombination and repair’ (1680, 8.72%). The following groups: ‘defense mechanism’ (73, 0.38%), ‘extracellular structure’ (15, 0.08%) and ‘nuclear structure’ (8, 0.04%) represented the smallest groups (Additional file 2: Figure S2).

### Expression and functional annotation of unigenes associated with ER stress-related pathway

**Figure 1:**
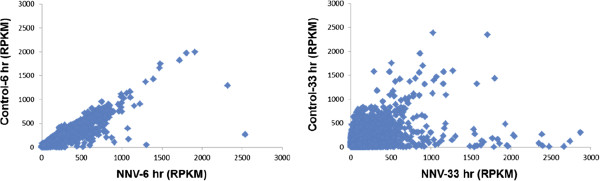
**Comparison of unigene expression among different DGE samples.** The gene expression level was calculated by using RPKM method (Reads Per kb per Million reads). As the RPKM method can eliminate the influence of different gene length and sequencing discrepancy on the calculation of gene expression, the calculated gene expression can be directly used for comparing the difference of gene expression among samples. The expression of unigenes in response to NNV infection was analyzed by comparing the RPKM of unigenes in the infected versus non-infected GK cells at 6 (A) and 33 (B) hours post-infection. NNV: NNV-infected GK cells, Control: non-infected GK cells.

The tag-based DGE analysis was performed to characterize the gene expression profile associated with NNV infection in GK cells. The comparison of unigene expression was measured by the RPKM (Reads Per kb per Million reads) method, which can be directly used for comparing the difference of gene expression among samples [24]. The RPKM value was compared between NNV-infected and non-infected cells at 6 and 33 hr post-infection. As shown in Figure 1, there was slight increase in the unigene expression in the NNV-infected sample at 6 hr post-infection, as indicated by the RPKM values scattering toward the X-axis (NNV-infected sample). At 33 hr post-infection, substantial numbers of unigenes were either up-regulated or down-regulated in the NNV-infected cells. To further characterize the biological functions that were affected by NNV, the 66,582 unigenes from the GK transcriptome database were subjected to analysis against Kyoto Encyclopedia of Genes and Genomes (KEGG) to assign their functions within known biological pathways. A total of 19505 unigenes were mapped into 218 KEGG pathways, among which 555 unigenes (2.85%) were related with the protein processing in ER.

**Figure 2:**
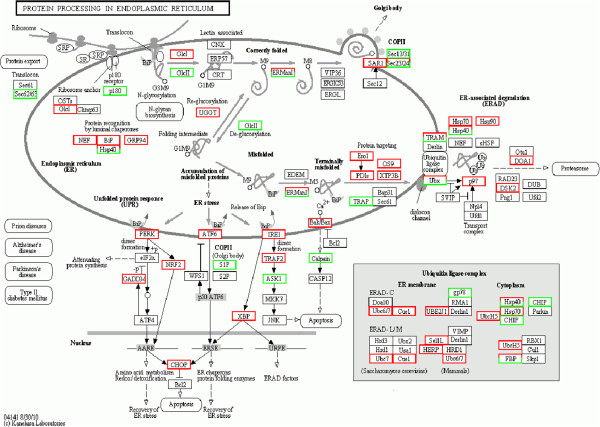
**Identification of unigenes relevant to protein processing in endoplasmic reticulum.** Analysis of the digital gene expression profile in NNV-infected GK cells at 33 hours post-infection identified a total of 117 unigenes that are assigned to genes known to be associated with protein processing in ER pathway. Red boxes refer to genes whose associated unigene(s) was up-regulated in the NNV-infected GK cells. Green boxes refer to genes whose associated unigene(s) was down-regulated. Boxes with both red and green colors indicate genes that might have isoforms.

**Figure 3:**
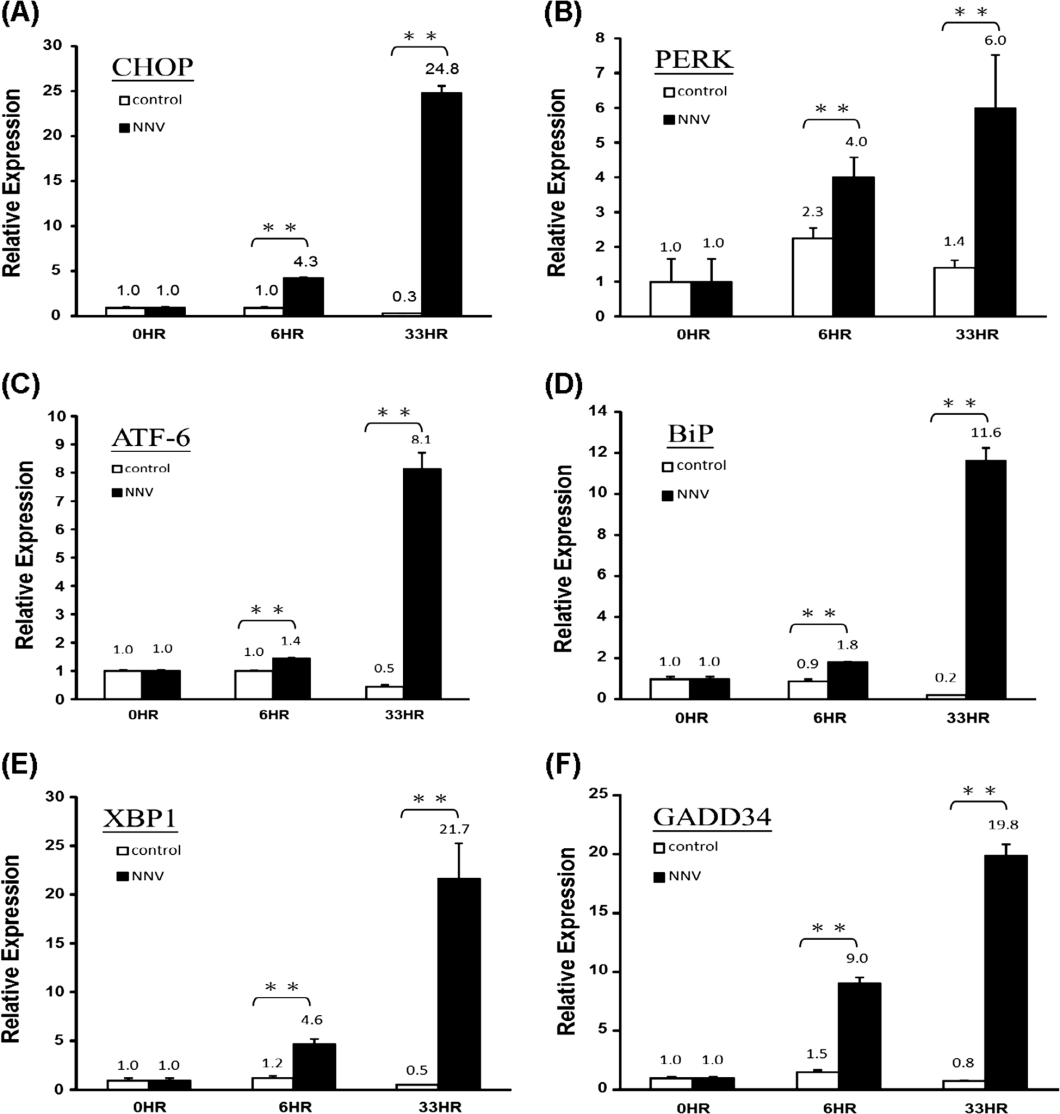
**Expression of CHOP, PERK, ATF6, BiP, XBP1 and GADD34 in NNV-infected GK cells.** GK cells were infected with NNV at MOI of 10, and harvested at 6 and 33 hr post-infection. Triplicate samples were collected. The expression level of CHOP, PERK, ATF6, BiP, XBP1 and GADD34 was measured by quantitative RT-PCR, and the relative expression of each gene was normalized against the expression level of β-actin. The average relative expression of each set of sample is indicated by the numbers above bars. The significance of difference was analyzed by Student’s *t*-test (*0.01 ≤ *p* ≤ 0.05; **p ≤ 0.01). Vertical bars represent the standard deviation (n = 3).

Analysis of the digital gene expression profile in NNV-infected GK cells revealed that the ER stress pathway was prominently affected by NNV infection (Figure 2). Out of the 555 ER-associated unigenes, a total of 117 unigenes were affected in NNV-infected GK cells. Among the genes associated with the 117 unigenes, 16 (related to 25 unigenes) are main players of the UPR and ER-associated degradation (ERAD) processes. These unigenes have been deposited to the NCBI database and are summarized with the assigned accession numbers in Table 2. At 33 hr post-infection, several major components of the UPR pathway were up-regulated, including BiP, PERK, ATF6, IRE1, GADD34, CHOP and XBP-1. Table 3 lists the fold of up-regulation of these genes. Among the genes, CHOP and GADD34, both are components of the PERK pathway, were also up-regulated at 6 hr post-infection. To verify the expression profile of these major UPR genes in response to NNV infection, we performed a time-course study by using quantitative RT-PCR analysis. As shown in Figure 3, GADD34 and CHOP were the two most affected genes at 6 hr post-infection, with 6-fold and 4.3-fold increase, respectively. Except for PERK, the relative expression of these genes at 33 hr post-infection detected by quantitative RT-PCR correlated well with the profile obtained by DGE analysis (Figure 3 and Table 3).

**Table 2:** Summary of unigenes relevant to ER stress identified in NNV-infected GK cells

**Gene**	**Function catagory**	**Unigene**	**Accession Number**	**Name in NCBI**
*CHOP*	UPR	45524	JR139431	DNA damage-inducible transcript 3 protein-like
		64294	JR139432	
*ATF6*	UPR	56294	JR139433	AMP-dependent transcription factor 6
*BiP*	UPR	44883	JR139434	Glucose-regulated protein 78 (GRP78/BiP)
		52488	JR139435	
*XBP1*	UPR	53059	JR139436	x-box binding protein 1
		63131	JR139437	
*IRE*	UPR	46736	JR139438	Endoplasmic reticulum to nucleus signaling 2
*TRAF2*	UPR	61284	JR139439	Tumor necrosis factor receptor-associated factor 2
		50968	JR13940	
*ASK1*	UPR	14083	JR13941	Mitogen-activaed protein kinase kinase kinase 5 (MAP3K5/ASK1)
*NRF2*	UPR	57155	JR13942	Nuclear factor erythroid 2-related factor 2
		64431	JR13943	
*GADD34*	UPR	62766	JX644070	Growth arrest and DNA damage-inducible protein/Protein phosphatase 1, regulatory subunit 15A
*GRP94*	Protein regulation by luminal chaperones	27245	JR13944	Glucose-regulated protein 94
*Hsp70*	ERAD	2073	JR13945	Heat shock protein 70
*Hsp90*	ERAD	54351	JR13946	Heat shock protein 90
*Ero1*	ERAD	26048	JR13947	Endoplasmic oxidoreductase-1-like protein
		27161	JR13948	
		40262	JR13949	
*PDIs*	ERAD	49848	JR13950	Protein disulfide-isomerase A6
		54206	JR13951	
		56611	JR13952	
*TRAP*	ERAD	50028	JR13953	Translocon-associated protein subunit alpha
*Bak/Bax*	ERAD	11469	JR13954	Apoptosis regulator BAX-like
*Calpain*	ERAD	59428	JR13955	Calpain-like protein

**Table 3:** Differential expression of ER-stress related unigenes in NNV infected GK cells at 6 and 33 hours post-infection

**Gene**	**Expression Level**	**6 Hours Fold Change**	**33 Hours Fold Change**
*CHOP*	Up	1.2	2.4
*PERK*	Up	NA	4.4
*ATF6*	Up	NA	1.2
*BiP*	Up	NA	3.0
*XBP1*	Up	NA	2.0
*GADD34*	Up	1.2	1.6

### Expression of BiP and the interaction between BiP and NNV capsid protein

**Figure 4:**
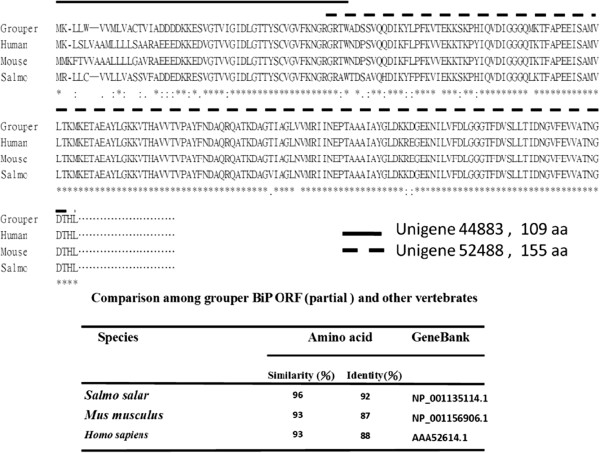
**Comparison of BiP amino sequence (partial) among grouper and other vertebrates.** Two unigenes (unigene44883 and unigene52488) of grouper BiP were obtained. Unigene44883 encodes the first 109 residues of the Bip open reading frame, and unigene52488 encodes 155 a.a., with 3 residues overlapped with the C’-terminus of the fragment of the first 109 a.a. A partial grouper BiP open reading frame showed greater than 93% and 87% in sequence similarity and identity with other vertebrates, respectively.

BiP is a master regulator of UPR response. The up-regulation of BiP in NNV-infected cells inspired us to investigate how BiP might interact with viral components in the infected cells. From our *de novo* sequencing data, we have identified two fragments of grouper BiP cDNA. The first fragment (unigene44883) represents the first 109 residues of the open reading frame, while the second fragment (unigene52488) is of 155 a.a., with 3 residues overlapped with the C’-terminus of the first fragment (Figure 4). A partial grouper BiP open reading frame obtained by compiling the two fragments showed greater than 93% and 87% in sequence similarity and identity with other vertebrate counterparts, respectively (Figure 4).

**Figure 5:**
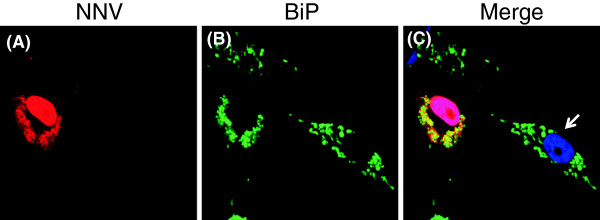
**Sub-cellular localization of NNV capsid protein and BiP protein in NNV-infected GF-1 cells.** Grouper fin cell line GF-1 was infected with NNV at MOI of 10. At 24 hr post-infection, the cells were fixed and co-stained with rabbit anti-NNV capsid and mouse anti-BiP, followed by Texas Red conjugated anti-rabbit IgG and Fluorscein conjugated mouse IgG. The Nucleus was visualized by counter-staining with 4', 6-diamidino-2-phenylindole (DAPI). The NNV capsid protein (**A**) is shown to co-localize with BiP protein (**B**) in distinct area within the cytoplasm as indicated by the merged image (**C**). Uninfected GF1 cell was indicated by white arrow.

The conservation in the amino sequence of BiP protein throughout evolution made it feasible for us to utilize commercially available BiP antibodies to further investigate the expression of BiP protein and its potential interaction with viral components. A series of functional assays were carried out with GF-1 cells, which has been popularly used as a model cell line for NNV infection [25]. As shown in Figure 5, the expression of BiP protein co-localized with the NNV capsid protein in the infected GF-1 cells. In non-infected cells, BiP protein formed as aggregated speckles dispersed throughout the cell cytoplasm, while in the infected cells, NNV capsid protein displayed both nuclear localization and a distribution pattern of interconnected web apart from perinuclear region. Furthermore, BiP protein in infected cells also exhibited punctuated pattern and strictly localized to the cytoplasmic area where capsid protein expressed. These results indicate an interaction between BiP and the viral capsid protein.

**Figure 6:**
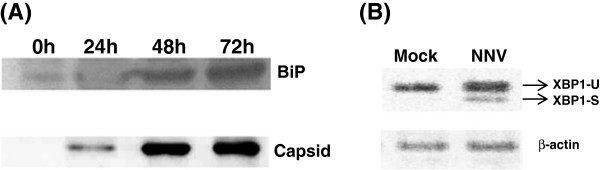
**Role of NNV capsid protein in ER stress.** (**A**) Co-immunoprecipitation of BiP protein and NNV capsid protein. GF-1 cells were infected with NNV at MOI of 5. The cell lysate was incubated with Protein A agarose and anti-BiP IgG. The immunocomplexes were separated on a 12% SDS-PAGE gel, and were further analyzed by western blotting with mouse anti-BiP and horseradish peroxidase-conjugated anti-mouse IgG. Chemiluminescence signal was captured after incubation with ECL substrate. For NNV capsid protein, the membrane was stripped, and re-probed with rabbit anti-NNV capsid antiserum, followed by horseradish peroxidase-conjugated anti-rabbit IgG. The result shows that BiP protein could interact with NNV capsid protein after NNV infection. (**B**) Induction of XBP1 mRNA splicing by NNV capsid protein *in vivo*. Zebrafish embryos at one-cell stage were mock-injected with 9.2 nl of sterile water or injected with 9.2 nl of pIR8 at the concentration of 150 ng/μl. The larvae were sacrificed at 20 hr post-fertilization and subjected to RNA extraction. RT- PCR was carried out to analyze the transcript of *xbp-1* gene. Splicing of XBP1 mRNA, an indicator of ER stress, was detected in the embryos expressing exogenous NNV capsid protein.

The interaction between BiP and NNV capsid protein was further confirmed by a co-immunoprecipitation assay, in which the capsid protein was co-precipitated with the BiP protein captured by anti-BiP antibodies (Figure 6A). This result strongly supports the notion of an interaction between BiP and NNV capsid protein. In addition, it may suggest a potential role of the NNV capsid protein in the ER stress response. We thus injected the NNV capsid gene into zebrafish embryos to evaluate the effect of capsid protein on ER stress *in vivo*. The splicing pattern of XBP-1 mRNA was measured as an indicator of the ER stress response. As shown in Fig 6B, exogenous expression of NNV capsid protein resulted in XBP-1 mRNA splicing as demonstrated by the production of XBP-1s. All together, these results support the notion that an interaction exists between NNV capsid protein and the UPR master regulator BiP, and that the interaction is associated with the ER stress response in the NNV-infected cells.

## Discussions

Grouper is an economically important fish species, whose life cycle is severely threatened by many pathogens, particularly viruses. NNV causes devastative mortality in grouper at the larvae and juvenile stages, and yet the understanding of its pathogenecity is limited, mainly hampered by the lack of proper tools and fundamental genomic information of the host. The leap of the NGS technology, such as Illumina Genome Analyzer, is a valuable tool providing high-volume, parallel sequencing throughput, thus extremely beneficial to the study of non-model species of no reference genomic resources, such as grouper [26]. The technology has been widely applied in gene discovery, digital gene expression profiling and comparative genomics studies [26-30]. In this study, we have employed the NGS technology to generate a transcriptome library of 66,582 unigenes from grouper GK cells. By using this library as a reference, we have identified in NNV-infected GK cells 117 unigenes representing genes that are known to be associated with protein processing in ER. The obtained sequence information will greatly assist in future studies on the molecular regulation of ER response in grouper cells under normal or pathological condition. Endoplasmic reticulum serves to maintain protein homeostasis, including the regulation of the concentration, conformation, folding, and trafficking of client protein in cells [31]. Many studies have revealed that the viral infection and replication of flavivirus family such as Japanese encephalitis virus, bovine viral diarrhea virus, and hepatitis C virus would induce ER dysfunction and stress [32-35]. Some other viruses such as respiratory syncytial virus, African swine fever virus, herpes simplex virus and cytomegalovirus have also been shown to regulate ER stress [36-39]. On the other hand, intracellular stress responses of UPR would induce apoptosis in cell if the ER stress is prolonged. Hence, the delicate modulation of UPR would determine the outcome of viral infection in host cells [40].

Recent studies have shown that ER stress plays a role in the apoptotic cell death caused by NNV infection [21]. However, the understanding of the molecular mechanism of NNV-induced ER stress response is limited. It has most recently been shown that BiP is up-regulated in grouper GF cells after NNV infection [21]. While the up-regulation of BiP after NNV infection is confirmed in this study, we have further shown that the three mediators (PERK, ATF6 and IRE1) controlled by BiP were up-regulated as well in the infected cells. Among the UPR-associated genes, CHOP and GADD34 were most prominently up-regulated at 6 hr post-infection. As both CHOP and GADD34 are components of the PERK pathway, the earlier up-regulation of these two genes suggests an earlier onset of the PERK pathway. This observation coincides with the notion that PERK functions at an earlier stage than ATF6 and IRE1 during ER stress response [17].

A surprising result of this study is that BiP protein may co-localize and interact with the capsid protein of NNV, as demonstrated by confocal microscopy and co-immunoprecipitation assays (Figures 5 &6A). Furthermore, overexpression of the capsid protein would trigger ER stress response *in vivo* (Figure 6B) and apoptotic cell death in the transfected cells (data not shown). Interestingly, it has been shown in a most recent study that BiP protein can interact with the RNA-dependent RNA polymerase (RdRp), and that the interaction of these two proteins may enhance mitochondria-mediated cell death [21]. Therefore, it appears that BiP protein may interact with at least two viral components (RdRp and capsid) in NNV-infected cells, and that the interaction between BiP and viral proteins is crucial to the outcome of NNV infection. Finally, we provide evidence that NNV capsid protein could enter the nucleus of infected cell during viral replication. This result indicates that NNV capsid protein might also play a role in the regulation of gene expression in the infected cells.

## Conclusions

In summary, we have established a database of genes that are involved in the ER stress response in NNV-infected grouper cells. Additionally, we have discovered a potential role of NNV capsid protein in the ER stress response. Another interesting discovery is that NNV capsid protein might play a role in the regulation of gene expression in infected cells as suggested by the presence of capsid protein in the nucleus. These data provide new insights into the molecular mechanism of NNV-induced ER stress, and will be of great value to future studies of NNV pathogenicity.

## Methods

### Cells and virus

The grouper GK cells [41] and GF-1 cells [25], were grown at 28°C in Leibovitz L-15 medium supplemented with 5% and 10% fetal bovine serum (FBS) (Invitrogen), respectively. Malabar grouper nervous necrosis virus (MGNNV) was propagated and titrated in GF-1 cells as described previously [42]. For viral infection, confluent cells in 6-well plates (Corning) were rinsed with serum-free L15 and infected with the virus at multiplicity of infection (MOI) of 5 (for GF-1 cells) or 10 (for GK cells), unless mentioned otherwise.

### RNA extraction

Total RNA was prepared by using Trizol® reagent (Invitrogen, USA) according to manufacturer’s instruction. Prior to Illumina sequencing, the quality of RNA was verified by Agilant 2100 Bioanalyzer (Agilent Technologies, USA) with a readout RIN value of 9.2, and the quantity of RNA was measured by using Nanodrop® ND-1000 spectrophotometer (LabTech, USA).

### Transcriptomic sequencing and reads assembly

Solexa sequencing was performed as a commercial service in Beijing Genomic Institute (BGI). After collection of total RNA, beads coated with Oligo(dT) were used to isolate poly(A) mRNA from the samples. First-strand cDNA was synthesized with random hexamer-primers. The second-strand cDNA was synthesized and purified. After purification, end reparation and adding poly(A), the cDNA fragments were tailed with sequencing adaptors. These fragments were then subjected to PCR amplification, and the products were subsequently sequenced by using Illumina HiSeq^™^ 2000. After sequencing, the SOAP *de novo* software [23] was used to assemble the data. The software first combined reads and overlaps to form longer fragments, called contigs. The contigs were further assembled into scaffolds by joining the paired-end reads of contigs. Gap filling of scaffolds by paired-end-reads was applied to obtained sequences with least Ns and not extendable on either ends. These sequences are known as unigenes.

### Unigenes annotation and functional classification

Homology searches of the assembled unigene sequences were conducted against public protein databases of Nr, Swiss-Prot, Kyoto Encyclopedia of Genes and Genomes (KEGG), and Cluster of Orthologous Groups (COG) by using blastx (the e-value was set to be < 0.00001). Highest sequence similarity of proteins were retrieved and assigned to specific unigene. Unigenes that cannot be aligned to any database were further scanned by ESTScan [43]. Functional annotation of unigenes was performed according to the KEGG Pathway, COG, and Gene Ontology (GO). GO was performed with non-redundant (nr) annotation by Blast2GO program [44].

### Comparison of unigene expression among different DGE samples

The gene expression level was calculated by using RPKM method (Reads Per kb per Million reads) [24], according to the formula: RPKM(A) = 10^5^C/(NL × 10^-3^). RPKM(A) stands for the expression of gene A, C for the number of reads that uniquely aligned to gene A, N for the total number of reads that uniquely aligned to all genes, and L for the number of bases on gene A. As the RPKM method can eliminate the influence of different gene length and sequencing discrepancy on the calculation of gene expression, the calculated gene expression can thus be directly used for comparing the difference of gene expression among samples. When there is more than one unigene for a gene, the longest one is used for the calculation of its expression level and coverage. The expression of unigenes in response to NNV infection was analyzed by comparing the RPKM of unigenes in the infected versus non-infected GK cells at 6 and 33 hours post-infection.

### Identification and expression profile of ER-stress related genes in NNV-infected GK cells

Unigenes that are relevant to ER stress were identified by the analysis against the KEGG Pathway. Quantitative RT-PCR was carried out to quantitate the expression level of selective ER-stress related genes: CHOP, PERK, ATF6, BiP, XBP1 and GADD34. The RNA was reverse-transcribed into cDNA using Superscript III Reverse Transcriptase from Invitrogen by following the manufacturer’s recommendation. Real-time PCR was performed with the reagent Express SYBR GreenER qPCR Supermix Universal (Invitrogen) by following the manufacturer’s suggestions. The reaction was carried out with triplicates of each sample on a Rotor-Gene Q thermal cycler (Qiagen). Relative expression of each gene was normalized against the house-keeping gene β-actin. The PCR primers are listed in Additional file 3: Table S1.

### Sub-cellular localization of NNV capsid protein and BiP

GF-1 cells were infected with NNV at MOI of 10 for 24 hours. Infected cells were fixed with 4% formaldehyde at room temperature for 15 minutes. Fixed GF-1 cells were then permeabilized with PBS containing 0.2% triton X-100, followed by blocking with 3% bovine serum albumin (BSA) in PBS for 1 hour. Indirect immunofluorescence to stain NNV coat protein and BiP was performed with rabbit anti-NNV capsid antibody [45] at 1:1000 dilution and mouse anti-BiP antibody (Santa Cruz, sc-166490) at 1:50 dilution. The secondary antibodies against rabbit IgG conjugated with Texas Red (Invitrogen, T2767) or antibodies against mouse IgG conjugated with Fluorscein (KPL, Cat.02-18-06) were diluted at 1:500. All antibodies were diluted in PBS containing 3% BSA. 4',6-diamidino-2-phenylindole (DAPI) was further applied to stained cells for nuclear visualization. Stained cells were mounted onto glass slides with PBS containing 0.1g/ml 1,4-Diazabicyclo- [2]octane (DABCO) (Sigma, D-2522), 50% glycerol, and 0.1% Na_3_N. Confocal images were acquired with Olympus FV1000 inverted confocal microscope.

### Co-immunoprecipitation assay

For co-immunoprecipitation (co-IP) assay, GF-1 cells at 90% confluency were infected with NNV (MOI = 5). The cells were harvested by trypsinization, washed three times with PBS, and lysed with cell lysis buffer (Cell Signaling, 9803S) at 0, 24, 48 and 72 hours post-infection. The cell lysate was collected after centrifugation at 14000 rpm for 20 min. The supernatant was precleared with Protein G-agarose beads (Sigma-Aldrich), and incubated overnight at 4°C with Protein A agarose (Invitrogen) and 2 μg anti-BiP IgG (Santa Cruz, sc-166490). The immunocomplexes were recovered by a brief centrifugation prior to separation on a 12% SDS–PAGE gel, and were subsequently transferred onto a nitrocellulose membrane. For BiP detection, the membranes were blocked with PBS-T containing 3% (w/v) non-fat milk (non-fat milk/PBS-T) for 1 h at room temperature. After washing, the membrane was incubated with mouse anti-BiP (1:1000, Santa Cruz) in 1% (w/v) non-fat milk/PBS-T overnight at 4°C. After blocking, the membrane was washed, and incubated with horseradish peroxidase-conjugated anti-mouse IgG (1:3000 in 0.1% PBS-T)(Zymed, 81–6520) for 1 h at room temperature. The membrane was incubated with ECL substrate (Pierce ECL Western Blotting Substrate, Thermo Scientific, 32209) for 1 minute, and visualized by a chemiluminescence image system (UVP, BioSpectrum Imaging System 500). For NNV capsid detection, the membrane was stripped with 20 ml of stripping buffer (Restore^™^ Western Blot Stripping Buffere, Thermo Scientific, 21059) at room temperature for 15 min, blocked with 3% (w/v) non-milk fat PBS-T at room temperature for 1 h, and re-probed with rabbit anti-NNV capsid antibody (1:3000 in 0.1% PBS-T) [46] overnight at 4°C. The membrane was incubated with secondary antibody horseradish peroxidase-conjugated anti-rabbit IgG (1:3000, PerkinElmar, NEF812001EA), and subsequently the ECL substrate.

### Plasmid and transfection

The NNV capsid protein gene was cloned into a eukaryotic expression vector pIRES-e2EGFP (Clontech) at the *Eco*RI-*Sal*I sites of the vector to generate a plasmid termed pIR8. The plasmid was transformed into JM109 cells and the transformed cells were grown in large quantity to isolate the plasmid. Isolation and purification of the pIR8 plasmid was conducted by using QIAprep Spin Miniprep Kit (Qiagen). Transfection of pIR8 was carried out with Fugene® 6 Transfection Reagent (Roche) by following the manufacturer’s instruction.

### Induction of XBP1 mRNA splicing by NNV capsid protein *in vivo*

Zebrafish embryos at one-cell stage were mock-injected or injected with 9.2 nl of pIR8 at the concentration of 150 ng/μl by using a microinjector (Leica EZ4 nanoliter 2000). The larvae were sacrificed at 20 hr post-fertilization and subjected to RNA extraction. The RNA was reversetranscribed to cDNA, followed by PCR analysis. The XBP-1 primers for the PCR are:5’-GTT CAG GTA CTG GAG TCC GC-3’ (forward primer) and 5’-GGA TGT CCA GAA TAC CAA GCA GG-3’ (reversed primer). The PCR product was analyzed by electrophoresis with a 3% agarose gel (UltraPure TM Agarose-1000, Invitrogen).

## Competing interests

The authors declare that they have no competing interests.

## Authors’ contributions

FHN and YMC carried out the NNV-induced ER stress experiments. NYC carried out the transcriptome and gene expression profiling experiments. FYL conducted the confocal microscopy experiment. MWL, YSL and PPC designed the experiments. MWL, FHN and PPC wrote the manuscript. All authors read and approved the final manuscript.

## Supplementary Material

Additional file 1 **Figure S1.** Histogram of gene ontology (GO) classification. Among the 66,582 unigenes, 76,975 unigenes were classified into the 51 sub-categories under the three categories of GO: molecular function, cellular component and biological process. Click here for file

Additional file 2 **Figure S2.** Histogram of clusters of orthologous groups (COG) functional classification. Out of the 66,582 unigenes, 19,261 unigenes were grouped into the 25 COG catagories. The largest group in COG is ‘General function prediction only’ (3673, 19.07%), followed by group ‘Transcription’ (1708, 8.87%) and group ‘Replication, recombination and repair’ (1680, 8.72%). Click here for file

Additional file 3 **Table S1.** Real-time PCR primers. Click here for file
