# Supplementary figures and images for: Transcriptome characterization and gene expression of Epinephelus spp in endoplasmic reticulum stress-related pathway during betanodavirus infection in vitro
Source: BMC Genomics. 2012 Nov 21;13:651. doi: 10.1186/1471-2164-13-651 (PMC3560219; doi:10.1186/1471-2164-13-651)

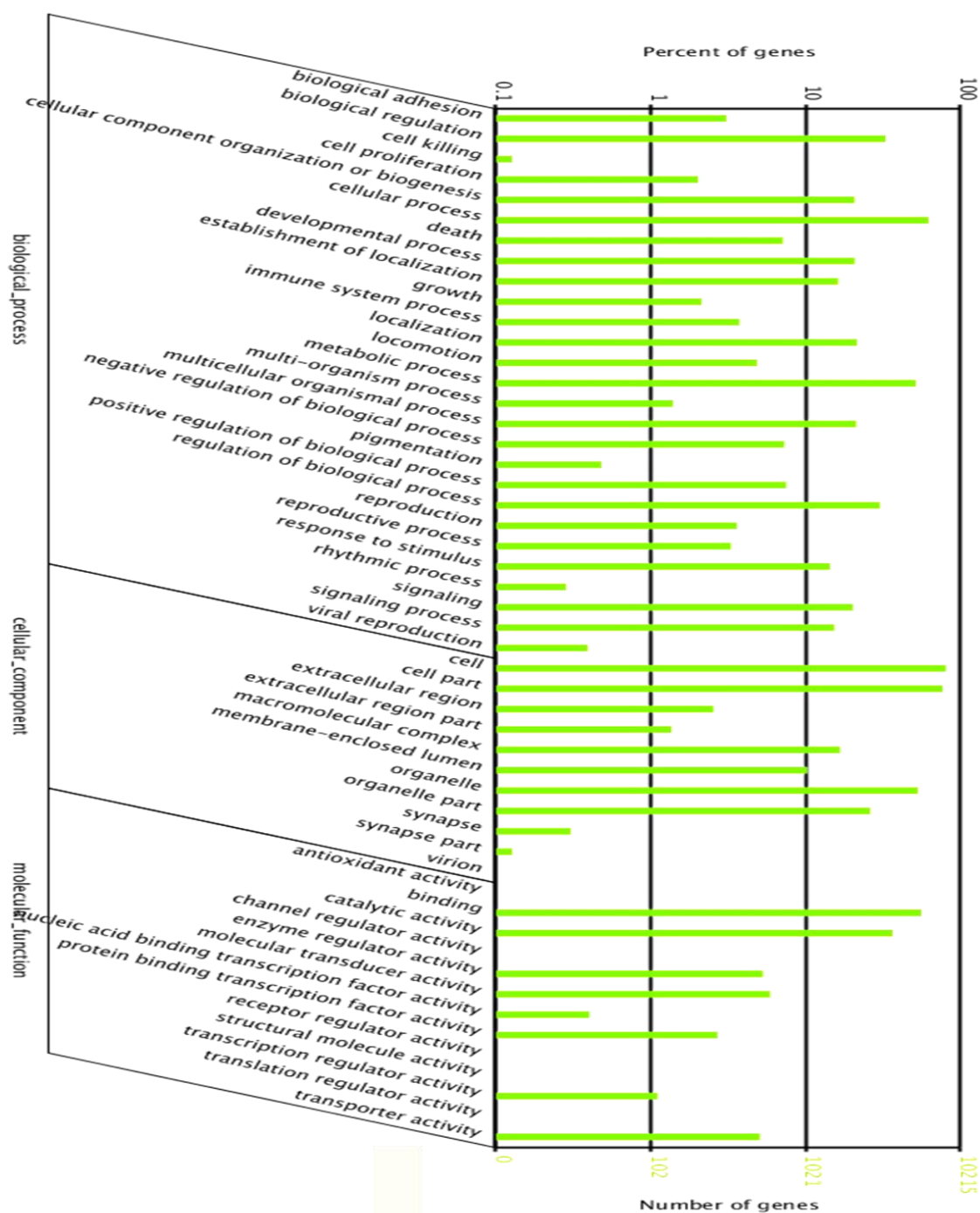

Fig. S1

Supplement: Additional file 1 — Figure S1. Histogram of gene ontology (GO) classification. Among the 66,582 unigenes, 76,975 unigenes were classified into the 51 sub-categories under the three categories of GO: molecular function, cellular component and biological process. [file 1471-2164-13-651-S1.pdf]

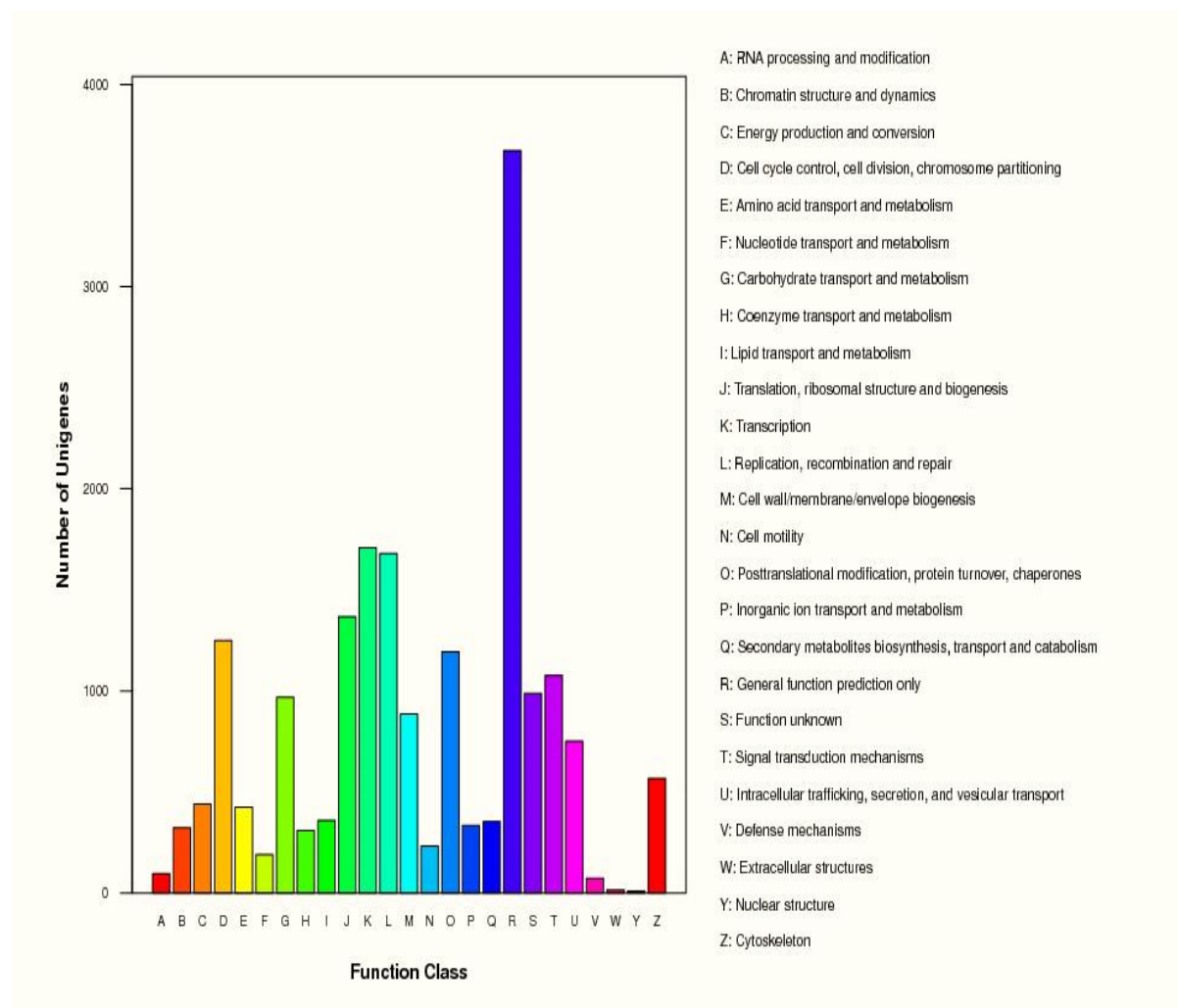

**Fig. S2**

Supplement: Additional file 2 — Figure S2. Histogram of clusters of orthologous groups (COG) functional classification. Out of the 66,582 unigenes, 19,261 unigenes were grouped into the 25 COG catagories. The largest group in COG is ‘General function prediction only’ (3673, 19.07%), followed by group ‘Transcription’ (1708, 8.87%) and group ‘Replication, recombination and repair’ (1680, 8.72%). [file 1471-2164-13-651-S2.pdf]
