# Supplementary material for: Transcriptome characterization and gene expression of Epinephelus spp in endoplasmic reticulum stress-related pathway during betanodavirus infection in vitro
Source: BMC Genomics. 2012 Nov 21;13:651. doi: 10.1186/1471-2164-13-651 (PMC3560219; doi:10.1186/1471-2164-13-651)
Supplement: Additional file 3 — Table S1. Real-time PCR primers. [file 1471-2164-13-651-S3.doc]

Table S1: Real-time PCR primers

Gene Primers

CHOP (F) 5’-TGGATGTTCTGGAGAGTTGTTCTC-3’

(R) 5’-TGTTGCCCTCTGGACACCTT-3’

AFTF6 (F) 5’-ATCCTCCACATCAGGTCTTCCA-3’

(R) 5’-GGCGAGGAGTGCTGGTAGAA-3’

PERK (F) 5’-GGACCAGAGGTGGCTGAAAG-3’

(R) 5’-TGACTGACAGCGCCTCCAT-3’

GADD34 (F) 5’-CACTGGTGCCCTGGAAAAGA-3’

(R) 5’-CTGGCTTGTGCTGACTTTGC-3’

BIP (F) 5’-GACCAGGGTAACCGCATCAC-3’

(R) 5’-TTCTTCGCAGCATCACCAATC-3’

XBP1 (F) 5’-AGGACGAGCCAGAGGAAGTG-3’

(R) 5’-GGAGGAGGTTTCGGAGAAGAA-3’

β-actin (F) 5’-CCTGACAGAGCGTGGCTACTC-3’

(R) 5’-CCTTGATGTCACGCACGATT-3’

F: forward primer; R: reverse primer
